# Comparative Effects of Recirculating and Rice-Co-Culture Systems on Growth-Quality Trade-Offs and Underlying Physiological Mechanisms in Red Claw Crayfish (*Cherax quadricarinatus*)

**DOI:** 10.3390/foods15111857

**Published:** 2026-05-24

**Authors:** Weiwei Lv, Zhiwei He, Weiwei Huang, Hang Yang, Quan Yuan, Yuning Zhang, Wenzong Zhou

**Affiliations:** 1 Eco-Environmental Protection Research Institute, Shanghai Academy of Agricultural Sciences, Shanghai 201403, China; wwlv@saas.sh.cn (W.L.); 18818266708@163.com (Z.H.); yhangqu2024@163.com (H.Y.); quanyuan2016@126.com (Q.Y.); ynzhang@saas.sh.cn (Y.Z.); 2 Key Laboratory of Integrated Rice-Fish Farming, Ministry of Agriculture and Rural Affairs, Shanghai Academy of Agricultural Sciences, Shanghai 201403, China; 3 College of Oceanography and Ecological Science, Shanghai Ocean University, Shanghai 201306, China; 4 Wuxi Fisheries College, Nanjing Agricultural University, Wuxi 214081, China

**Keywords:** red claw crayfish, recirculating aquaculture systems, rice–crayfish co-culture, muscle quality, intestinal microbiota, metabolic adaptation

## Abstract

The red claw crayfish (*Cherax quadricarinatus*) is an economically important freshwater crustacean. This study comprehensively compared the physiological profiles of crayfish cultured in recirculating aquaculture systems (RAS) and rice–red claw crayfish co-culture systems (RRCS) over a 92-day experimental period (3 replicates per system; *n* = 15 crayfish sampled per group), focusing on growth performance, muscle quality, hepatopancreatic metabolomics, and intestinal microbiota. Results demonstrated that while RAS provided a significant growth advantage, RRCS exhibited superior muscle quality characterized by higher protein, lower moisture, and firmer texture. Regarding intestinal microecology, RAS induced a microbial shift toward the potentially pathogenic genus *Vibrio*, whereas RRCS promoted core symbionts like *Candidatus Hepatoplasma*. Multi-omics integration revealed that RRCS altered the intestinal microbiota and was associated with up-regulated hepatopancreatic pantothenate biosynthesis and TCA cycle pathways, alongside LysoPC-mediated membrane lipid remodeling. In conclusion, while RAS promotes rapid somatic growth, RRCS fosters a distinct intestinal microecology and hepatopancreatic metabolic profile that aligns with enhanced muscle quality. These findings provide a theoretical foundation for optimizing ecological aquaculture models.

## 1. Introduction

The red claw crayfish (*Cherax quadricarinatus*) is one of the most popular and premium freshwater crustaceans globally. Its muscle is exceptionally rich in high-quality protein and n−3 polyunsaturated fatty acids (PUFAs), notably eicosapentaenoic acid (EPA) and docosahexaenoic acid (DHA), establishing it as a highly valuable freshwater food source with significant commercial prospects [1,2]. According to FAO statistics, the annual production in traditional major producing regions, such as Australia and Southeast Asia, remains limited to approximately 260 tons [3]. In contrast, this industry has expanded rapidly in China, with annual production exceeding 20,000 tons and market prices for premium individuals reaching up to 20 USD/kg [4]. Given its surging industrial and nutritional significance, systematically evaluating the nutritional quality and edible properties of red claw crayfish under different culture models is of paramount importance.

Currently, red claw crayfish aquaculture predominantly relies on earthen pond culture [5]. Although technically mature, this method is characterized by unstable yields and vulnerability to climatic fluctuations [6]. To meet the demands of industrial upgrading, two distinct innovative aquaculture paradigms have emerged. The rice–red claw crayfish co-culture system (RRCS) represents an ecological approach, utilizing shallow paddy fields to construct diversified habitats that ensure optimal water quality and abundant natural forage, such as benthic organisms [7,8]. Extensive empirical studies confirm the positive regulatory effects of integrated paddy habitats on the quality of aquatic products. For instance, in *Procambarus clarkii* co-culture systems, habitat heterogeneity and natural prey intake significantly enhance muscle essential fatty acids (EFAs) and hepatopancreatic free amino acids [9]. Furthermore, rice–fish (*Pelteobagrus fulvidraco*) systems can substantially enrich beneficial gut microbiota, thereby comprehensively improving host metabolic health [10]. Conversely, the recirculating aquaculture system (RAS) follows an industrialized route [11]. Through precise environmental control, RAS maximizes growth rates and volumetric production yields [12].

The influence of the rearing environment on aquatic animals extends beyond macroscopic growth rates, profoundly manifested in their health status, muscle quality (texture and nutrition), and intrinsic physiological and metabolic characteristics [13,14]. Fundamentally, the deposition of flavor compounds and nutritional components in muscle is the outcome of the digestion, absorption, and metabolic conversion of environmental resources by the organism under a specific health state [15,16]. The intestine and hepatopancreas play pivotal roles in the organism’s digestive, immune, and metabolic processes. The intestinal microecology not only determines nutrient acquisition efficiency through the secretion of digestive enzymes but also maintains immune homeostasis via its biological barrier function [17], thereby mitigating the energy expenditure required to resist opportunistic pathogens [18]. Meanwhile, as the metabolic regulatory center, the core function of the hepatopancreas lies in balancing the resource allocation between “immune defense” and “growth deposition” [19,20]. When the organism is in a healthy metabolic state, the hepatopancreatic metabolic pathways shift from “anti-stress and detoxification” towards “anabolism”, thereby significantly promoting the efficient deposition of proteins and flavor compounds in the muscle [18]. While the paddy ecological environment is known to synergistically ameliorate intestinal and hepatopancreatic health in aquatic animals [21], a critical research gap remains: to date, it is unclear how growth-oriented RAS and quality-oriented RRCS differentially regulate these interconnected physiological processes and how such internal shifts ultimately drive the formation of distinct muscle quality profiles in red claw crayfish.

Therefore, we hypothesize that the RRCS environment fosters a healthier intestinal microecology and induces a beneficial metabolic shift, collectively driving a resource reallocation that favors superior muscle quality. To test this hypothesis, we applied a multi-omics approach—a strategy that has yielded significant insights in aquatic species such as yellow catfish and tilapia [10,21]. We systematically evaluated the growth performance, muscle quality, digestive enzymes, intestinal microbiota, and hepatopancreatic metabolomics of red claw crayfish under RAS and RRCS to provide a theoretical foundation for optimizing ecological aquaculture.

## 2. Materials and Methods

### 2.1. Experimental Site and Climatic Conditions

This study was conducted from May to August 2025, involving two aquaculture models: RAS and RRCS. Both experiments were carried out at the Zhuanghang Comprehensive Experiment Station of the Shanghai Academy of Agricultural Sciences (30°53′28″ N, 121°23′22″ E). This location has a typical subtropical monsoon climate. During the experimental period, the average monthly temperature was approximately 27.4 ± 3.1 °C, with an average monthly precipitation of 495.2 ± 25.5 mm.

### 2.2. Experimental Design and Farming Protocols

#### 2.2.1. Experimental Animals and Acclimation

The red claw crayfish used in this study were obtained from the Shanghai Xiangsheng Aquaculture Professional Cooperative. Prior to the formal experiment, all individuals underwent a 7-day acclimation period in an indoor RAS at the Zhuanghang Comprehensive Experiment Station of the Shanghai Academy of Agricultural Sciences. During the acclimation period, the crayfish were maintained in concrete tanks (3.0 m × 5.0 m × 0.5 m) and fed ad libitum with commercial pelleted feed daily to maintain their nutritional status. The animals were fasted for 24 h before grouping.

#### 2.2.2. Rice–Red Claw Crayfish Co-Culture System (RRCS)

The RRCS experiment was conducted in standardized paddy fields. Each experimental unit covered an area of 667 m^2^, with three independent paddy fields serving as biological replicates. A U-shaped peripheral trench was excavated around each field as the primary culture area, accounting for approximately 12% of the total area, while the remaining 88% consisted of the rice planting platform (80%) and the ridge area (8%). The trench was approximately 1.0 m deep, with a 30 cm clay seepage-proof layer at the bottom. Ridges were reinforced to a height of 60 cm and covered with high-density polyethylene (HDPE) film. Metal mesh covers with a 0.25 mm aperture were installed at the inlets and outlets to prevent predator entry and crayfish escape. To facilitate the free movement of crayfish between the paddy field and the trench, polyvinyl chloride (PVC) pipes (10 cm in diameter) were embedded every 2 m in the ridges as biological corridors.

Rice (*Oryza sativa* L.) planting and crayfish stocking were performed according to a specific timeline. Rice seedlings were transplanted in late May with a spacing of 25 cm × 25 cm. Submerged macrophytes, including *Vallisneria natans* and *Elodea nuttallii*, were planted in the culture trenches with 35% vegetation coverage to construct a quasi-natural ecosystem, providing natural plant food and shelter for the crayfish [22]. Red claw crayfish juveniles (9.05 ± 0.2 g) were stocked in July at a density of 37,500 ind/ha. The culture period lasted until harvest in September.

Unified fertilization and feeding strategies were applied during the experiment. A basal-fertilizer-oriented strategy was adopted for rice cultivation, with the formula including 550 kg/ha urea, 600 kg/ha superphosphate, and 120 kg/ha potassium chloride. Regarding aquaculture management, commercial feed containing 36.0% crude protein, 4.0% crude lipid, and 8.0% crude fiber was provided daily in the late afternoon (17:00). A dynamic adjustment method was used for the feeding strategy, with the daily ration controlled at 3–5% of the total crayfish biomass. No pesticides or herbicides were used throughout the experiment to ensure the ecological safety of the co-culture system.

#### 2.2.3. Recirculating Aquaculture System (RAS)

The RAS experiment was conducted in a controlled indoor environment. Three independent culture tanks (0.785 m^2^ × 1.0 m) were utilized as biological replicates for the RAS group. Juvenile crayfish were stocked at a density of 19.1 ind/m^2^ (15 individuals per tank). Each unit was equipped with an independent recirculating filtration system consisting of a submersible pump, PVC conduits, and a filtration box filled with zeolite and biological sponges. The system operated for 2 h daily for water purification and biological filtration. To mimic the natural habitat of the red claw crayfish and mitigate intraspecific aggression, sufficient PVC pipes and artificial refuges for crayfish were provided at the bottom of the tanks.

The photoperiod was strictly controlled at 14 h of light and 10 h of darkness (14L:10D). Water quality management involved a combined strategy of intermittent circulation and periodic water exchange: the filtration system operated for 2 h daily to maintain water purity [23], and 25% of the total water volume was replaced every 14 days to ensure the long-term stability of water quality parameters [24]. For daily management, a standardized feeding strategy was adopted, with commercial feed (containing 36.0% crude protein, 4.0% crude lipid, and 8.0% crude fiber) provided daily at 17:00. The daily ration was controlled at 3–5% of the total biomass. To minimize feed loss and facilitate ingestion, the circulation pumps were temporarily deactivated during feeding. Residual feed and feces were removed promptly after feeding before restarting the system.

#### 2.2.4. Water Quality Monitoring

To ensure the comparability and reliability of the experimental models, the water quality of both the RAS and RRCS was rigorously monitored. Basic parameters, including water temperature, pH levels, and dissolved oxygen (DO), were assessed in situ five times daily using a portable water quality analyzer (model HQ40D, HACH, Loveland, CO, USA). Furthermore, water samples were collected weekly to quantify ammonia nitrogen (NH_4_^+^-N), nitrite nitrogen (NO_2_^−^-N), and chemical oxygen demand (COD) levels using standard chemical methods. As shown in Table 1, regular monitoring confirmed that water quality in both the RAS and RRCS was consistently maintained within the safe physiological ranges for red claw crayfish [25,26,27].

### 2.3. Sample Collection

After 92 days, all red claw crayfish were fasted for 24 h and anesthetized in an ice bath. A random sampling strategy was employed, whereby five healthy and appendage-intact individuals (*n* = 15 per group) were randomly captured from each experimental unit. Their body weight and carapace length were measured to evaluate growth performance. Following these measurements, the sampled individuals were divided into two subgroups for subsequent analyses: three individuals (*n* = 9 per group) were dedicated to muscle texture profile analysis (TPA) to ensure muscle structural integrity; the remaining two individuals (*n* = 6 per group) were dissected aseptically under low-temperature and sterile conditions. For the anatomical samples, the foregut and a portion of the hepatopancreas were first collected, flash-frozen in liquid nitrogen, and stored at −80 °C for digestive enzyme activity assays. Another portion of the hepatopancreas was similarly flash-frozen and stored at −80 °C for untargeted metabolomic analysis to reveal lipid and amino acid metabolic characteristics. Subsequently, the hindgut was aseptically isolated, and its contents were carefully collected and stored at −80 °C for 16S rRNA intestinal microbial community sequencing. Finally, the abdominal muscle was completely stripped and stored at −40 °C for proximate composition analysis. To ensure strict data traceability, each crayfish was assigned a unique alphanumeric ID at capture (e.g., RAS_01–RAS_15, RRCS_01–RRCS_15). All tissues (hepatopancreas, hindgut contents, muscle) and measurements (body weight, carapace length) from the same individual were labeled with that ID using a consistent suffix system (e.g., ‘-H’ for hepatopancreas, ‘-G’ for gut, ‘-M’ for muscle). Specific subsets (*n* = 15 for growth, *n* = 9 for texture, *n* = 6 for microbiota and metabolomics) were used for different analyses, and within each subgroup individual IDs were maintained throughout all processing and data recording steps.

### 2.4. Sample Analysis

#### 2.4.1. Growth Performance and Morphophysiological Indices

This study analyzed the red claw crayfish from two dimensions: growth performance and morphophysiological characteristics. In addition to traditional growth rate and survival indicators, such as weight gain rate (WGR), specific growth rate (SGR), and survival rate (SR), this study focused on the organosomatic indices of the crayfish. Specifically, the hepatosomatic index (HSI) was utilized as a crucial parameter to evaluate the energy reserves and metabolic status of the hepatopancreas. Meanwhile, the condition factor (CF) and meat yield (MY) were used to characterize the overall plumpness and muscle development of the organism. The specific calculation formulas are as follows:

$$\mathrm{Weight}\;\mathrm{gain}\;\mathrm{rate}:\;WGR\;(\%)=\frac{W_{t}-W_{0}}{W_{0}}\times 100$$

$$\mathrm{Specific}\;\mathrm{growth}\;\mathrm{rate}:\;SGR\left(\%\right)=\frac{lnW_{t}-lnW_{0}}{T}\times 100$$

$$\mathrm{Survival}\;\mathrm{rate}:\;SR\;(\%)=\frac{N_{t}}{N_{0}}\times 100$$

$$\mathrm{Hepatosomatic}\;\mathrm{index}:\;HSI\;(\%)=\frac{W_{hep}}{W_{t}}\times 100$$

$$\mathrm{Meat}\;\mathrm{yield}:\;MY\;(\%)=\frac{W_{mus}}{W_{t}}\times 100$$

$$\mathrm{Condition}\;\mathrm{factor}:\;CF\;(g/{cm}^{3})=\frac{W_{t}}{L^{3}}\times 100$$

where W_0_ and W_t_ represent the initial and final body weights (g) of the red claw crayfish, respectively; T is the duration of the experiment (92 days); N_0_ and N_t_ represent the initial number and the final number of surviving individuals, respectively; W_hep_ and W_mus_ refer to the hepatopancreas weight (g) and muscle weight (g), respectively; and L represents the carapace length (cm).

#### 2.4.2. Measurement of Digestive Enzyme Activities

Tissues (foregut and hepatopancreas) were rinsed with ice-cold phosphate-buffered saline (PBS) and homogenized in saline (1:10, *w*/*v*). Following homogenization, the samples were centrifuged at 4 °C (4000 rpm, 10 min) to collect the supernatants. These extracts were subsequently analyzed for digestive enzyme profiles (cellulase, lipase, trypsin, and amylase) using commercial kits (Nanjing Jiancheng Bioengineering Institute Co., Ltd., Nanjing, China). These commercial diagnostic kits have been widely and reliably employed for enzymatic assays across various decapod crustaceans, including closely related crayfish models [28].

#### 2.4.3. Muscle Quality Analysis

TPA was performed on cubic dorsal muscle samples (1 cm^3^) using a TA.XT Plus texture analyzer (Stable Micro Systems, Godalming, UK). The instrument was equipped with a cylindrical flat-ended probe to execute a double-compression test at ambient temperature. Specific settings included a fixed strain of 30% deformation, a resting period of 5 s between cycles, and probe speeds of 2.0 mm/s (pre-test) and 1.0 mm/s (test). Key mechanical attributes, such as hardness, cohesiveness, springiness, resilience, gumminess, and chewiness, were calculated from the resulting force-time curves.

#### 2.4.4. Muscle Proximate and Nutritional Composition

The chemical composition of muscle tissue (moisture, crude protein, crude lipid, and ash) was evaluated following standard protocols [29]. Specifically, moisture and ash contents were quantified gravimetrically by subjecting samples to thermal treatment at 105 °C (drying) and 550 °C (ashing for 6 h), respectively. For crude components, the Kjeldahl method (N × 6.25) and Folch’s extraction (using a chloroform/methanol solvent system) were utilized to determine protein and lipid levels [30].

To analyze the amino acid profile, approximately 50 mg of the muscle sample was hydrolyzed in 6 mol/L HCl at 110 °C for 22 h. After the hydrolysate was filtered through a 0.45 µm membrane, it was analyzed using an Agilent 1200 high-performance liquid chromatography (HPLC) system (Agilent Technologies, Santa Clara, CA, USA) equipped with an online OPA-FMOC pre-column derivatization system (Column: Agilent ZORBAX Eclipse XDB-C18) [31].

The fatty acid profile was characterized via gas chromatography–mass spectrometry (GC-MS; Agilent 7890B, Santa Clara, CA, USA). Briefly, muscle samples (0.5 g) were homogenized in a 1:2 (*v*/*v*) methanol/chloroform solution (5 mL). Following filtration and the addition of 4 mL deionized water, the mixture was centrifuged (2300× *g*, 5 min, 4 °C) to isolate the lower lipid layer. The extracted lipids were dissolved in 1 mL of *n*-hexane and subjected to methyl esterification using 1 mL of 0.4 mol/L KOH-methanol for 30 min. Finally, phase separation was achieved with deionized water (2 mL), and the upper organic layer was injected for analysis. Quantification relied on the area normalization method [32].

#### 2.4.5. 16S rRNA Gene Sequencing and Bacterial Community Analysis

Total genomic DNA was extracted from the intestinal content samples (*n* = 6 per group, 12 samples in total) using the MagBeads FastDNA Kit for Soil (MP Biomedicals, Santa Ana, CA, USA). Following quality control and concentration quantification via a NanoDrop NC2000 spectrophotometer (Thermo Fisher Scientific, Waltham, MA, USA) spectrophotometer and 1% agarose gel electrophoresis, the V3–V4 region of the bacterial 16S rRNA gene was amplified using the sample-specific barcode-indexed primers 338F and 806R [33,34]. The purified and equimolarly pooled amplicons were sequenced on the Illumina NovaSeq platform (2 × 250 bp paired-end; Illumina, Inc., San Diego, CA, USA) by Shanghai Personal Biotechnology Co., Ltd. (Shanghai, China).

Raw sequence data were processed using QIIME2 (v2024.5). The DADA2 plugin was utilized for quality filtering (quality threshold Q > 30), denoising, merging, and chimera removal to generate amplicon sequence variants (ASVs). Taxonomic assignment was performed against the actively curated SILVA 138 database (99% similarity), selected for its comprehensive sequence coverage and superior taxonomic resolution compared to legacy databases [35,36,37]. Downstream bioinformatic analyses of the intestinal microbiota, including alpha diversity, beta diversity (evaluated via PCoA and NMDS based on Bray–Curtis distances), taxonomic composition, and functional prediction, were conducted using the online Personal Cloud Platform (www.genescloud.cn). Microbial functional potential was predicted from ASV abundances using PICRUSt2, and KEGG Orthologs were compared between groups.

#### 2.4.6. LC-MS Analysis for Metabolomics

The metabolite extraction procedure was as follows: first, 30 mg of the sample was weighed into a centrifuge tube, and 200 μL of pre-chilled deionized water was added for the sample to be ground and homogenized. Subsequently, 800 μL of a methanol/acetonitrile mixture (1:1, *v*/*v*) was added. After ultrasonic extraction for 30 min, the mixture was left to precipitate at −20 °C for 45 min. Following cold centrifugation (12,000 rpm, 4 °C, 20 min), 800 μL of the supernatant was collected and vacuum-dried. The residue was reconstituted in 150 μL of 50% methanol containing an internal standard (L-2-chlorophenylalanine). After a second centrifugation (12,000 rpm, 4 °C, 10 min), the supernatant was filtered through a 0.22 μm membrane prior to analysis. A quality control (QC) sample was prepared by pooling 10 μL aliquots from each filtered sample.

Chromatographic separation was performed using a Thermo Vanquish Flex UHPLC system (Thermo Fisher Scientific, Waltham, MA, USA) equipped with an ACQUITY UPLC HSS T3 column (2.1 × 100 mm, 1.8 μm; Waters Corp., Milford, MA, USA). The column temperature was maintained at 40 °C, and the flow rate was set to 0.4 mL/min. Mobile phase A consisted of a 0.1% formic acid aqueous solution, and mobile phase B was acetonitrile containing 0.1% formic acid. Separation was achieved using a gradient elution program.

Mass spectrometry detection was conducted on a Thermo Orbitrap Exploris 120 mass spectrometer (Thermo Fisher Scientific, Waltham, MA, USA) equipped with a heated electrospray ionization (HESI) source. The spray voltages were set to 3.5 kV and −3.0 kV for the positive and negative ion modes, respectively. The ion source parameters were set as follows: sheath gas, 40 arb; auxiliary gas, 10 arb; capillary temperature, 320 °C; and auxiliary gas heater temperature, 300 °C. Data acquisition was performed in data-dependent acquisition (DDA) mode with a scan range of 70–1000 m/z. The full MS resolution was set to 60,000, and the MS/MS resolution was set to 15,000. The top four precursor ions with the highest response intensities were selected for higher-energy collisional dissociation (HCD) fragmentation at a normalized collision energy of 30%. The dynamic exclusion time was set to 45 s. Raw MS data were processed using MS-DIAL (v4.9.221218) for peak extraction, alignment, and gap-filling. Metabolite identification was validated according to the Metabolomics Standards Initiative (MSI) guidelines. The differential metabolites were putatively annotated at MSI Level 2 (probable structure) by matching retention time, accurate precursor mass (mass tolerance of 0.01 Da/mass error < 10 ppm), and MS/MS fragmentation patterns (mass tolerance of 0.05 Da) against the PSNGM database (integrating mzCloud, HMDB, and LIPID MAPS) and an in-house library, with an identification score cutoff of 70. The detailed MSI confidence level criteria are provided in Supplementary Table S1. Quality control was performed using the pooled QC sample injected every 10 runs. The total ion chromatograms (TIC) of QC samples showed consistent retention times and peak intensities (Supplementary Figure S1), pairwise correlation coefficients among QC samples were >0.99 (Supplementary Figure S2), and more than 90% of metabolite features had RSD < 30% (Supplementary Figure S3). Features with RSD > 30% were excluded from downstream analysis.

### 2.5. Statistical Analysis

Data computation was performed using Microsoft Excel 2019, and statistical analysis was performed using SPSS 27.0. Prior to parametric analyses, the normality of data distribution and homogeneity of variance were verified using the Shapiro–Wilk and Levene’s tests, respectively. Differences between the two groups were evaluated using the independent samples *t*-test or the Mann–Whitney U test (for non-parametric data). For multiple comparisons across numerous non-metabolomic functional modules (e.g., growth metrics, texture profile parameters, proximate composition, specific amino acids, and fatty acids), the Benjamini–Hochberg False Discovery Rate (FDR) procedure was systematically applied to adjust the initial *p*-values, generating the corresponding q-values to strictly control for Type I errors. Statistical significance was declared at an FDR-adjusted q < 0.05. Results are presented as mean ± SD. Multivariate analyses (PCA, OPLS-DA) and Pearson correlation were performed via MetaboAnalyst 6.0. Differential metabolites (VIP > 1.0 and FDR < 0.05) were mapped to the KEGG database for pathway enrichment (hypergeometric test, *p* < 0.05).

## 3. Results

### 3.1. Growth Performance

As shown in Table 2, there were no significant differences in the W_0_ and SR of the red claw crayfish between the two groups (*p* > 0.05), indicating consistent initial experimental conditions and comparable survival across both culture modes. However, the RAS group exhibited markedly enhanced growth performance and morphophysiological indices compared to the RRCS group. Specifically, the W_t_, WGR, SGR, HSI, MY, and CF were all significantly elevated in the RAS group (*p* < 0.001).

### 3.2. Digestive Enzyme Activities

**Figure 1:**
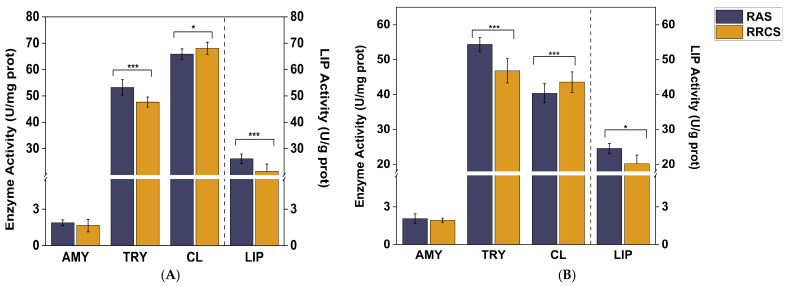
Effects of two different culture modes on digestive enzyme activity levels in the intestine (**A**) and hepatopancreas (**B**) of red claw crayfish (*n* = 9). Asterisks indicate significant differences between the groups (*, FDR q < 0.05, ***, FDR q < 0.001). Furthermore, large effect sizes (Cohen’s d > 0.8) were observed for the significant differences between the RAS and RRCS groups. In the intestine: Trypsin (d = 2.24), Lipase (d = 2.01), and Cellulase (d = 1.05). In the hepatopancreas: Trypsin (d = 2.67), Lipase (d = 2.16), and Cellulase (d = 1.14).

As shown in Figure 1A,B, culture systems significantly affected digestive enzyme activities in red claw crayfish. The RAS group displayed pronounced elevation of trypsin (TRY) and lipase (LIP) activities in both the hepatopancreas and intestine than the RRCS group (FDR q < 0.001), whereas the RRCS group showed markedly higher cellulase (CL) activity (FDR q < 0.05). However, no significant difference was observed in amylase activity between the two groups (FDR q > 0.05).

### 3.3. Effects of Different Culture Modes on Muscle Quality

#### 3.3.1. Muscle Textural Properties

Table 3 presents the muscle textural properties evaluated via TPA. The results demonstrated that the culture mode significantly influenced the muscle texture of the red claw crayfish. Compared to the RAS group, individuals in the RRCS group displayed superior values across all measured textural parameters, including hardness, springiness, resilience, cohesiveness, gumminess, and chewiness (*p* < 0.05).

#### 3.3.2. Proximate Composition of Muscle

As shown in Table 4, compared with the RAS group, the RRCS group showed marked elevation of crude protein and crude lipid in red claw crayfish muscle (*p* < 0.05), while the moisture and ash contents were substantially lower (*p* < 0.05).

#### 3.3.3. Amino Acid Profile of Muscle

As shown in Table 5, compared with the RAS group, the RRCS group exhibited pronounced increases in total amino acids (TAA), essential amino acids (EAA), and non-essential amino acids (NEAA) in muscle (*p* < 0.001). Except for lysine (Lys), which showed no significant difference, the contents of other major essential amino acids (such as Leu, Ile, and Val) and individual non-essential amino acids were significantly greater in the RRCS group, and the content of delicious amino acids (DAA) was also higher.

#### 3.3.4. Fatty Acid Profile of Muscle

As shown in Table 6, the muscle fatty acid composition of red claw crayfish differed significantly between the two culture systems. The contents of saturated fatty acids (SFA) and PUFA precursors (mainly C18 series) were markedly elevated in the RAS group than in the RRCS group (*p* < 0.01). In contrast, the contents of monounsaturated fatty acids (MUFA) and long-chain polyunsaturated fatty acids (LC-PUFA) were substantially higher in the RRCS group. Specifically, although the total PUFA content in the RRCS group was lower than that in the RAS group, the RRCS group demonstrated pronounced increases in ARA, EPA, DHA, and the n−3/n−6 ratio (*p* < 0.05).

### 3.4. Effects of Different Culture Modes on Intestinal Microbiota Diversity

#### 3.4.1. Assessment of the Intestinal Microbiota via 16S rRNA Gene Sequencing

**Figure 2:**
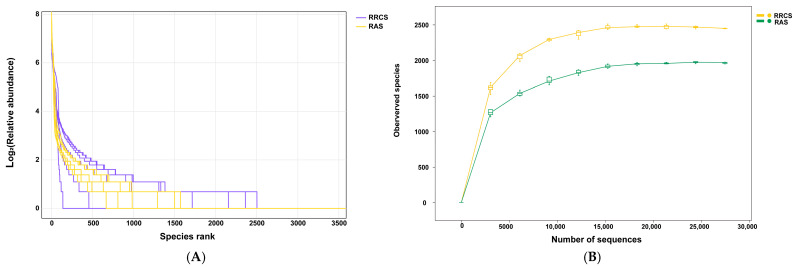
Rank-abundance and rarefaction curves of red claw crayfish under different farming modes. (**A**) Rank-abundance curves. (**B**) Rarefaction curves.

As illustrated in Figure 2A, the rank-abundance curves for all treatment groups exhibited a wide span on the X-axis and a gentle downward slope, indicating high species richness and an even distribution within the intestinal microbial communities of the red claw crayfish. Furthermore, the rarefaction curves (Figure 2B) gradually plateaued with increasing sequencing depth, demonstrating that the current sequencing depth was saturated and sufficient to capture the vast majority of microbial taxa within the samples.

#### 3.4.2. Alpha Diversity Analysis of the Intestinal Microbiota

**Figure 3:**
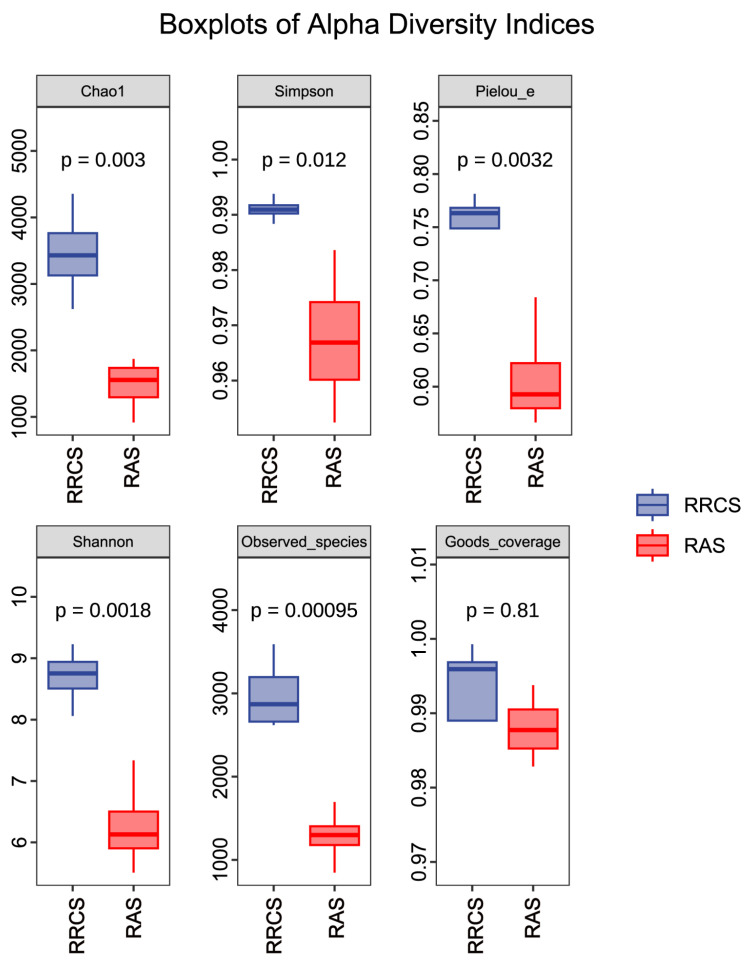
Alpha diversity indices of the intestinal microbiota in red claw crayfish between two farming modes. Box plots showing Observed species, Chao1, Shannon, Simpson, Pielou’s evenness, and Good’s coverage indices. Blue: RRCS; Red: RAS.

As shown in Figure 3, Good’s coverage indices of all samples were greater than 0.96, with no significant difference between the two groups (*p* > 0.05), indicating that the sequencing depth was sufficient to cover the vast majority of microbial taxa in the samples. Further α-diversity analysis revealed that the species richness indices (Observed species, Chao1), diversity indices (Shannon, Simpson), and evenness index (Pielou’s evenness) in the RRCS group were significantly higher than those in the RAS group (*p* < 0.01).

#### 3.4.3. Beta Diversity Analysis of the Intestinal Microbiota

**Figure 4:**
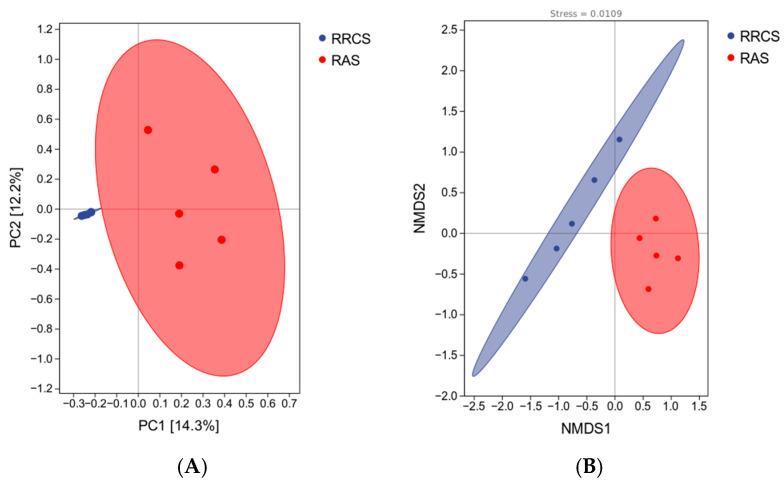
Beta diversity analysis of the intestinal microbiota in red claw crayfish. (**A**) PCoA plot (Bray–Curtis distances). (**B**) NMDS plot (Stress = 0.0109). Blue: RRCS; Red: RAS. Ellipses indicate 95% confidence intervals. PERMANOVA based on Bray–Curtis distances (999 permutations) further confirmed a significant difference in microbial community structure between the two groups (F = 1.35, R^2^ = 0.1193, *p* = 0.001).

Figure 4 illustrates the PCoA and NMDS plots based on Bray–Curtis distances, aimed at evaluating the differences in the beta diversity of the intestinal microbial communities of red claw crayfish under different culture modes. The PCoA results (Figure 4A) revealed that samples from the RRCS and RAS groups exhibited distinctly separated clustering patterns in their spatial distribution, featuring clear intergroup boundaries with no overlap. These findings were highly consistent with the NMDS analysis (Figure 4B), where the exceptionally low stress value (0.0109) indicated an excellent goodness of fit for the ordination model.

#### 3.4.4. Taxonomic Composition of the Intestinal Microbiota

To further investigate the effects of different culture systems on the intestinal microbiota structure of red claw crayfish, the species composition and relative abundance at the phylum and genus levels were analyzed in all samples.

**Figure 5:**
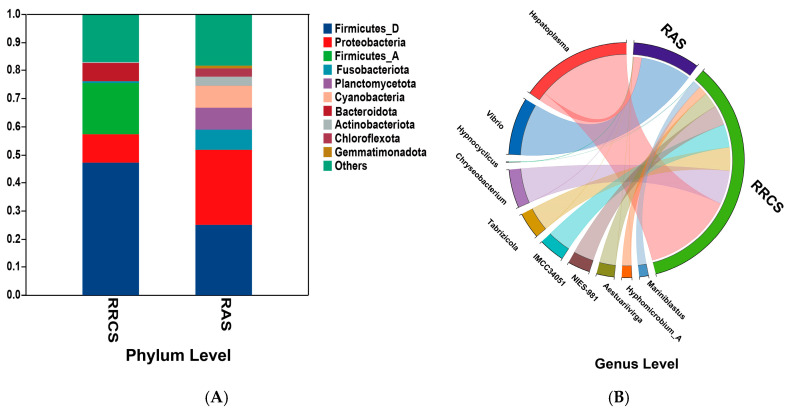
Composition and distribution of intestinal microbiota under different farming modes. (**A**) Stacked bar charts of relative abundance at the phylum level. (**B**) Chord diagram at the genus level revealing specific associations.

As shown in Figure 5A, the intestinal microbiota structure differed significantly between the two groups at the phylum level. *Firmicutes* and *Bacteroidetes* were the dominant phyla in the RRCS group. In contrast, the community structure in the RAS group shifted markedly, with a significantly increased relative abundance of Proteobacteria and decreased relative abundance of other phyla.

Further analysis at the genus level (Figure 5B) revealed that the elevated abundance of Proteobacteria in the RAS group was mainly attributed to a significant increase in the relative abundance of *Vibrio*. In contrast, the RRCS group exhibited a low abundance of *Vibrio*, with Hepatoplasma as the core dominant genus. In addition, the abundance of *Chryseobacterium* was higher in the intestine of the RRCS group. Detailed quantitative relative abundances of the dominant bacterial phyla and genera across all samples are provided in Supplementary Tables S3 and S4, respectively.

#### 3.4.5. Functional Prediction of the Intestinal Microbiota

**Figure 6:**
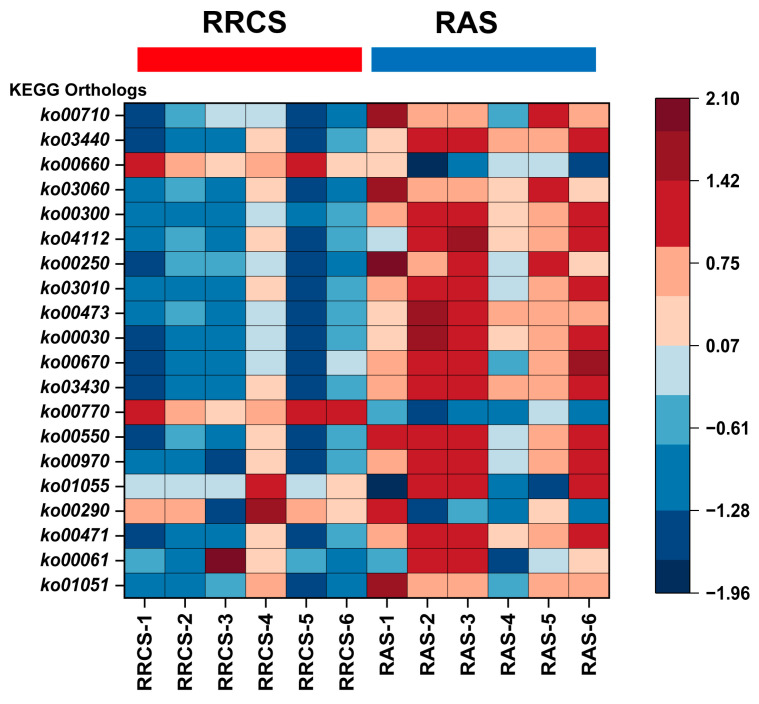
Functional divergence of the intestinal microbiota predicted by PICRUSt2.

PICRUSt2 analysis revealed distinct functional profiles between the two groups (Figure 6). The RRCS-associated microbiome was characterized by significantly higher predicted abundances of ‘Pantothenate and CoA biosynthesis’ (*ko00770*) and ‘Valine, leucine and isoleucine biosynthesis’ (*ko00290*). Notably, the Z-score standardized abundances of *ko00770* showed complete separation, with positive values in all six RRCS samples and negative values in all six RAS samples. Conversely, the RAS-associated microbiome exhibited significantly higher predicted abundances of pathways related to DNA repair (e.g., ‘Mismatch repair’, *ko03430*; ‘Homologous recombination’, *ko03440*) and bacterial cell wall synthesis (e.g., ‘Peptidoglycan biosynthesis’, *ko00550*; ‘D-Alanine metabolism’, *ko00473*; ‘D-Glutamine and D-glutamate metabolism’, *ko00471*).

### 3.5. Hepatopancreatic Metabolomics Analysis of Red Claw Crayfish Under Different Culture Modes

#### 3.5.1. Multivariate Analysis and Identification of Differential Metabolites

**Figure 7:**
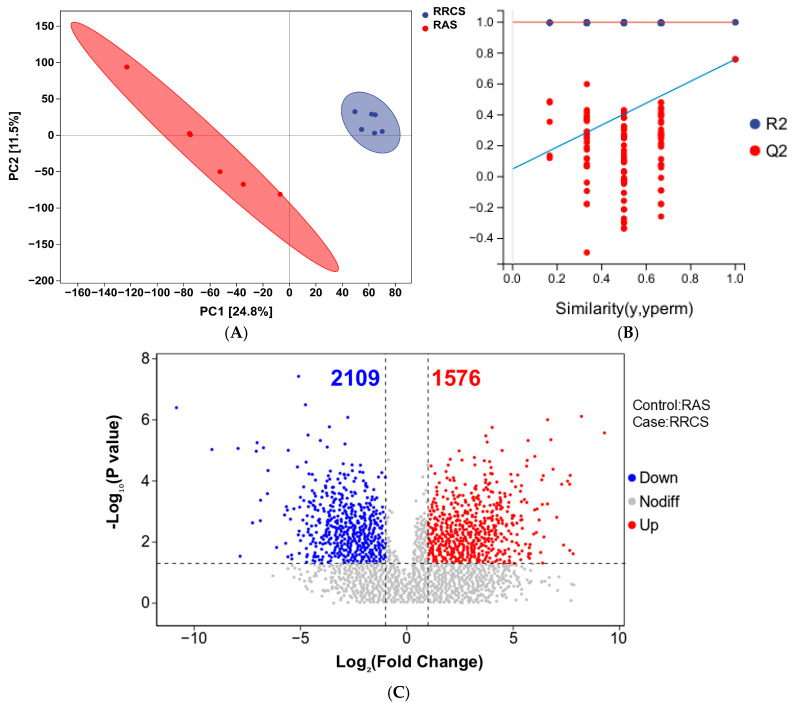
Analysis of hepatopancreatic metabolic profiles. (**A**) PCA analysis of hepatopancreatic metabolites. (**B**) OPLS-DA score plot showing separation between RAS and RRCS groups (R^2^Y = 1.000, Q^2^ = 0.726). (**C**) Volcano plot analysis of differential hepatopancreatic metabolites.

Untargeted metabolomics was used to analyze the metabolic profiles of the hepatopancreas in red claw crayfish under different culture systems. The PCA results revealed a distinct intergroup separation between the two groups, indicating significant differences in their metabolic profiles (Figure 7A). The OPLS-DA model further confirmed the significant separation between the groups (Figure 7B), and the model exhibited excellent robustness and predictive ability (R^2^Y = 1.000, Q^2^ = 0.726). Based on the criteria of VIP > 1.0 and FDR < 0.05, a total of 3685 significantly differentially expressed metabolites (DEMs) were identified. Compared with the RAS group, 1576 metabolites were significantly up-regulated, and 2109 metabolites were significantly down-regulated in the RRCS group (Figure 7C).

#### 3.5.2. Metabolic Correlation and Pathway Enrichment

**Figure 8:**
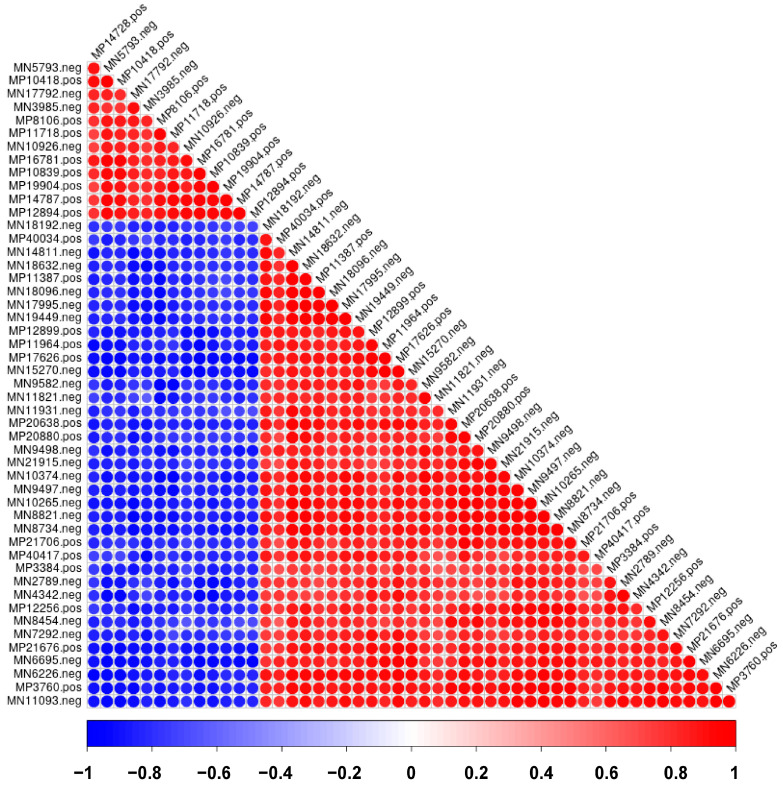
Correlation analysis of differentially abundant metabolites in the hepatopancreas of red claw crayfish under different aquaculture modes. Red represents positive correlation, and blue represents negative correlation.

To explore the synergistic response patterns of the metabolic networks under different culture modes, a correlation analysis of the differentially abundant metabolites was conducted using Pearson correlation coefficients (Figure 8, Table S2). In the heatmap, red and blue denote positive and negative correlations, respectively, and the color intensity reflects the correlation strength (statistical significance was set at *p* < 0.05).

The analysis revealed clear modular clustering characteristics among the metabolites. Specifically, dipeptides (represented by Valylalanine, Thr-His, Phe-Trp, and Arg-Phe) and lipids/secondary metabolites (such as LysoPE(22:4), Soudanone C, Bufol, and Grosheimin) each formed a tight cluster with strong intra-group positive correlations. Notably, a highly significant negative (antagonistic) correlation was observed between these two major metabolic clusters. This indicates that when adapting to differential environments, the red claw crayfish undergoes an essential metabolic divergence between amino acid metabolism and lipid metabolism, optimizing the allocation of energy and material resources to prioritize critical physiological demands under specific conditions.

**Figure 9:**
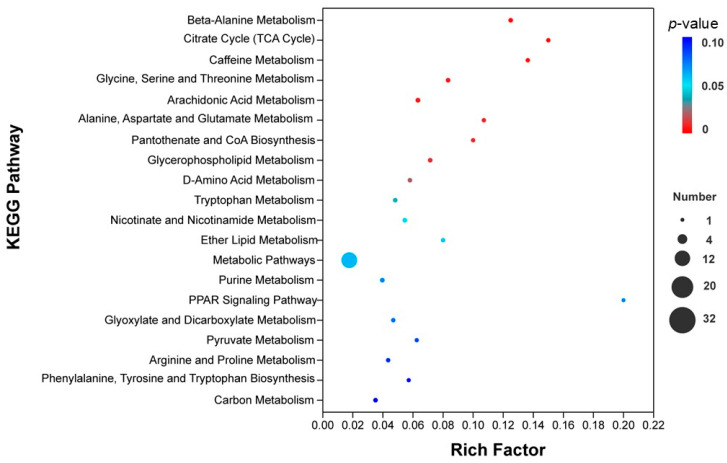
KEGG pathway enrichment analysis of differential metabolic pathways in the hepatopancreas of red claw crayfish under different aquaculture modes. The size of the bubble represents the number of metabolites, and the color indicates the *p*-value.

To systematically elucidate the regulatory pathways of different culture modes on the hepatopancreatic metabolic functions of the red claw crayfish, pathway enrichment analysis was performed based on the KEGG database (Figure 9, Table S3). Using a hypergeometric test with a significance threshold of *p* < 0.05, the differentially abundant metabolites between the RRCS and RAS groups were primarily enriched in the top 10 key metabolic pathways, including β-alanine metabolism (4 metabolites), citrate cycle (TCA cycle) (3 metabolites), caffeine metabolism (3 metabolites), glycine, serine and threonine metabolism (4 metabolites), arachidonic acid metabolism (5 metabolites), alanine, aspartate and glutamate metabolism (3 metabolites), pantothenate and CoA biosynthesis (3 metabolites), glycerophospholipid metabolism (4 metabolites), D-amino acid metabolism (4 metabolites), and tryptophan metabolism (4 metabolites) (Figure 9, Table S3).

In-depth qualitative analysis of these enriched pathways revealed an overall activation state of the hepatopancreatic metabolic network under the RRCS mode. Among the 37 differential metabolites identified within these 10 key pathways, 30 (approximately 81%) were significantly up-regulated in the RRCS group. Specifically, energy metabolism and cofactor biosynthesis were significantly enhanced, as evidenced by the significant accumulation of TCA cycle intermediates (citrate, fumarate, and L-malate) and coenzyme A synthesis precursors (pantothenate, 4′-phosphopantothenate, and uracil). Concurrently, key molecules involved in membrane lipid remodeling and signal transduction, such as lysophosphatidylcholines (LysoPCs, e.g., LysoPC 22:4 and LysoPC 22:5) and prostaglandin H2, were also significantly up-regulated. In contrast, only a few metabolites, such as carnosine, cadaverine, and colfosceril palmitate, were specifically down-regulated.

## 4. Discussion

The present study found that the red claw crayfish in the RAS mode displayed markedly enhanced growth performance (W_t_, WGR, and SGR) compared to those in the RRCS mode. Based on bioenergetic principles, it is hypothesized that this growth advantage is primarily attributed to the stable environmental conditions of the RAS, which effectively reduced the maintenance metabolic costs of the organism [38,39]. In contrast, the more expansive space and complex benthic habitats in the RRCS group necessitated the allocation of more energy toward physical activities, thereby reducing the energy share available for overall biomass expansion to some extent [40,41]. At the morphophysiological level, the RAS group showed significantly elevated hepatosomatic index (HSI), meat yield (MY), and condition factor (CF) (*p* < 0.001). The elevated HSI in the RAS group likely reflects excessive energy intake and hepatopancreatic lipid deposition, which may in turn promote rapid somatic growth and biomass accumulation, as reflected by the higher MY and CF [42]. This interpretation is supported by studies showing that excessive energy intake can lead to hepatopancreatic lipid accumulation in crustaceans [43,44]. However, this growth advantage appears to be achieved at the expense of muscle quality, as evidenced by lower crude protein, higher moisture, and inferior texture in RAS muscle (Table 4, Table 5 and Table 6).

The differences in digestive enzyme activities offer a plausible physiological basis for the significant divergence in growth performance mentioned above. The results showed that the activities of trypsin and lipase in the intestine and hepatopancreas of the RAS group were significantly higher than those of the RRCS group (*p* < 0.001). This phenomenon is highly consistent with the findings of Thirunavukkarasar et al. [45] regarding the “substrate adaptation” of digestive enzymes in aquatic animals. That study indicated that the expression of digestive enzymes exhibits significant plasticity: higher protein and lipid levels in formulated feeds act as substrate signals, inducing the organism to specifically up-regulate the expression of proteases and lipases. This highly active digestive enzyme system likely enhanced the nutrient assimilation efficiency of the organism, potentially providing sufficient metabolic substrates for rapid somatic cell proliferation and biomass accumulation, thereby promoting the accelerated growth observed in the RAS group [46]. In contrast, the RRCS group demonstrated enhanced cellulase activity (*p* < 0.05). This reflects the adaptive physiological regulation of red claw crayfish to cellulose-rich natural food sources (such as aquatic plants) in the rice–crayfish co-culture environment; such adaptive environmental enzyme regulation has also been reported in studies of other crustaceans [47]. However, limited by the relatively low nutrient utilization efficiency of plant-based diets [48], the RRCS group, despite possessing a stronger capacity for cellulose digestion, still exhibited lower overall growth performance than the RAS group, which was primarily supported by high-energy formulated feeds. It should be noted that the two culture systems inherently differ in spatial scale, habitat complexity, and water volume. To ensure meaningful comparison, all controllable factors (initial body weight, feed formulation, feeding rate, and experimental duration) were strictly standardized.

Analysis of the proximate composition of the muscle revealed that the red claw crayfish in the RRCS group exhibited distinct characteristics of “low moisture, high protein, and high lipid” content. This result is highly consistent with previous findings regarding red swamp crayfish (*Procambarus clarkii*) and common carp (*Cyprinus carpio*) reared in integrated rice–aquaculture systems [49,50]. Compared to the RAS group, which relies primarily on formulated feeds, the intake of abundant natural food sources in the RRCS has been shown to effectively promote the accumulation of muscle dry matter and enhance energy density in aquatic animals [51]. This “concentration effect” of nutrients not only improves the nutritional value of the muscle but also potentially contributes to its firmness and macroscopic textural characteristics. The superior muscle texture and higher crude protein in RRCS crayfish, despite a lower growth rate, point to a differential energy allocation strategy, likely driven by two synergistic factors. First, the extended nutrient accumulation period in ecological farming significantly improves muscle cellularity and texture, a phenomenon similarly observed in loach [52]. Second, the complex paddy ecosystem necessitates active foraging. Such exercise-induced remodeling is known to actively promote protein deposition and likely contributed to the enhanced muscle hardness observed in this study [53]. Together, these factors effectively counteract the flesh-softening effects associated with the rapid, high-energy-driven growth in the RAS group.

Amino acid composition is a core factor determining the nutritional value and sensory quality of aquatic products [54]. The present study demonstrated that the total contents of EAAs and DAAs in the muscle of the RRCS group were markedly elevated compared to those in the RAS group. This finding is highly consistent with the research results of Zhao et al. [30] and Wang et al. [21] in yellow swamp eel and tilapia, which suggests that compared to industrial intensive culture modes, ecological culture models (such as rice–fish co-culture) tend to better promote the accumulation of flavor substances in the muscle of aquatic organisms. Specifically, glutamic acid (Glu), glycine (Gly), and alanine (Ala) are widely recognized as the core contributors to the “umami and sweet” characteristic flavors of aquatic products [55]. The enrichment of these key flavor precursors in the RRCS group may be attributed to the ingestion of protein-rich benthos and plankton within the rice-field ecosystem. This diversified dietary pattern has been proven to effectively expand the free amino acid pool in the muscle of aquatic animals [9], thereby conferring superior potential flavor characteristics to the product.

Fatty acid composition is a key indicator for evaluating the nutritional value and human health benefits of aquatic products [56]. Although the RAS group possessed a higher total PUFA content, its primary components were C18-series precursor fatty acids. In contrast, the RRCS group exhibited superior lipid nutritional quality, characterized by lower SFA content and significant enrichment of MUFA and highly bioactive LC-PUFAs. Epidemiological studies have shown that reducing SFA intake while increasing unsaturated fatty acid intake helps prevent cardiovascular diseases [57]. In this study, the RRCS mode significantly increased the n−3/n−6 ratio in the muscle, making it more aligned with the dietary requirements for modern human cardiovascular health [58]. This finding is highly consistent with existing literature, which indicates that ecological culture modes are often superior to intensive recirculating aquaculture systems in optimizing fatty acid balance [9,30,59]. This advantage primarily appears to stem from the natural food web (such as algae and plankton) providing richer lipid sources than traditional commercial feeds. Specifically, the EPA and DHA enriched in algae have been shown to effectively optimize the lipid composition of muscle [60,61].

The diversity and stability of the intestinal microbial community constitute a critical defense line for maintaining the immune homeostasis and health of the red claw crayfish [62]. In the present study, the RRCS group exhibited significantly greater r α-diversity and a distinct community separation pattern (β-diversity) compared to the RAS group. Previous studies on rice–aquaculture systems have confirmed that diversified environmental input is key to breaking the microecological bottlenecks of artificial farming [63]. Our results further suggest that, compared to the highly artificial and simplified feeding in the RAS mode, the RRCS mode leverages the coupling effect of the “habitat-food web” to provide irreplaceable natural food sources and exogenous microbial inputs. This diversified nutritional and microbial component likely broadens the intestinal niches and constructs a more robust intestinal microecosystem by enhancing functional complementarity among the microbiota [64].

Further taxonomic composition analysis showed that the intestinal microbiota of red claw crayfish in the RRCS group was dominated by *Firmicutes* and *Bacteroidetes*. This is consistent with the findings of Wang et al. [21] in a rice–fish symbiotic system, indicating that the complex ecological environment of paddy fields can significantly enhance the species richness of the intestinal microbiota in aquatic organisms. Within *Firmicutes*, *Clostridium* can decompose polysaccharides to produce short-chain fatty acids (SCFAs), such as butyrate, which provide energy for intestinal epithelial cells and inhibit pathogen colonization by maintaining microecological balance. Meanwhile, *Bacteroidetes* utilize diverse hydrolase systems to degrade dietary fiber, assisting the host in achieving efficient nutrient utilization in complex environments [65].

A pivotal finding of this study lies in the succession of core genera and the divergence in microecological risks. Under the RAS mode, the potential pathogenic risks in the intestinal microecosystem increased, as evidenced by the significant enrichment of Proteobacteria and the potentially pathogenic genus *Vibrio* [19,62,66,67]. This phenomenon is consistent with findings in European eel RASs [68]. Such microecological shifts are often closely related to the closed nature of intensive farming systems, the uniformity of feed sources, and continuous environmental stress, which collectively may allow pathogenic microorganisms to occupy favorable ecological niches [66].

In contrast, the RRCS mode constructed a significant ecological defense barrier for the red claw crayfish. In this mode, the crayfish ingested large amounts of natural complex food from the paddy field (such as plant detritus) [69], and the significantly elevated cellulase activity confirmed the effect of this natural diet. It is plausible that this fiber-rich diet reshaped the substrate metabolic environment within the intestine, providing exclusive nutritional niches for specific symbiotic bacteria [70]. Based on this habitat filtering and diet-driven mechanism, the core symbiont *Candidatus Hepatoplasma* became the dominant colonizer in the RRCS environment [7]. Along with the extensive proliferation of this symbiotic group, opportunistic pathogens such as *Vibrio* may have been deprived of survival space due to “competitive exclusion” [67]. This microecological reorganization, driven by a natural diet, fundamentally enhances the environmental resilience and overall health of the red claw crayfish.

The differences in the culture environment were associated with metabolic adjustment in the hepatopancreas of the crustaceans [71]. The present study showed that the “pantothenate and CoA biosynthesis” pathway (involving pantothenate and 4′-phosphopantetheine) and the TCA cycle (citrate, malate, etc.) were significantly up-regulated in the RRCS group. As a key precursor of coenzyme A, the enrichment of pantothenate suggests an improvement in mitochondrial bioenergetic efficiency, which may provide the core drive for high-intensity protein synthesis and deposition in the organism [72].

Regarding lipid metabolism, the RRCS group exhibited distinct membrane lipid remodeling characteristics, specifically manifested by the substantial up-regulation of glycerophospholipid metabolites such as the LysoPC series. The optimization of this phospholipid configuration is hypothesized to not only enhance the organism’s resilience to environmental fluctuations by maintaining cell membrane fluidity but also help improve the transmembrane transport efficiency of nutrients [30]. Combined with the significantly reduced cadaverine load in the RRCS group, this microecological homeostasis implies lower levels of gut-derived toxins and pathogenic pressure [73]. This is highly consistent with previous conclusions that rice–prawn systems can effectively inhibit opportunistic pathogens and reshape healthy intestinal communities [70]. Consequently, this may reflect a metabolic shift in the RRCS group, potentially redirecting resources from “immune defense consumption” toward “muscle structural optimization and densification at the microscopic level, while simultaneously consuming energy for environmental exploration at the macroscopic level. Furthermore, the significantly active tryptophan metabolism reflects the host’s active adaptation to the complex paddy field habitat [74]. The accumulation of β-alanine, an anti-fatigue molecule [75], may be related to the increased physical activity caused by the expansive space of the paddy field [76]. The synergy between the environmentally induced “exercise effect” and high-flux anabolism likely contributed to the superior muscle textural properties of the red claw crayfish within the RRCS [77].

From the perspective of the gut-hepatopancreas axis, this study provides insights into the potential metabolic interaction network associated with how the culture environment may reshape the physiological quality of red claw crayfish. Multi-omics integration analysis suggests that the enrichment of *Chryseobacterium* in the gut of the RRCS group is significantly positively correlated with the hepatopancreatic pantothenate content and the TCA cycle pathway. This suggests that the core intestinal microbiota, while improving the bioavailability of natural food sources in the paddy field, also may provide a critical precursor pool for the host’s metabolic network [78]. This gut-driven bioenergetic priming effect is thought to provide sufficient metabolic momentum for efficient protein deposition in the muscle, thereby providing a plausible interpretation for the significantly higher total protein and essential amino acid (EAA) contents in the muscle of the RRCS group.

The PICRUSt2 functional predictions provide cross-omics support for the observed host metabolic divergence. The RRCS-associated microbiome was enriched in pantothenate and CoA biosynthesis (*ko00770*) [79], a pathway that supplies the essential cofactor for mitochondrial acetyl-CoA metabolism and fatty acid oxidation—functions that align with the elevated hepatopancreatic pantothenate levels and TCA cycle activation (Figure 6). Similarly, enrichment of branched-chain amino acid biosynthesis (*ko00290*) [80], which generates valine, leucine, and isoleucine as substrates for muscle protein synthesis, parallels the higher BCAA content in RRCS muscle (Table 5). Conversely, the RAS microbiota showed elevated DNA mismatch repair (*ko03430*) [81] and homologous recombination (*ko03440*) [82]—pathways induced by replication stress—as well as peptidoglycan biosynthesis (*ko00550*, *ko00471*, *ko00473*) [83,84,85], a signature of active cell wall turnover in Gram-negative bacteria. These functional signatures are consistent with Vibrio proliferation and the environmental stress of intensive recirculating systems. Although PICRUSt2 remains a predictive tool requiring future metagenomic validation, the coherence between inferred microbial functions and host multi-omics phenotype strengthens the gut-hepatopancreas-muscle axis interpretation.

Furthermore, the succession of the intestinal microbiota plays a key role as an “immunometabolic checkpoint” in the transition of the body’s health homeostasis [86]. The recruitment of *Candidatus Hepatoplasma* and the subsidence of the pathogen *Vibrio* in the RRCS group are hypothesized to have reduced the transport of gut-derived endogenous toxins (such as cadaverine) to the hepatopancreas. This low-stress gut-hepatopancreas communication mode likely prompted an adaptive redirection of the metabolic flux in the hepatopancreas [87,88]: shifting from a “stress-defense”-prioritized consumption mode in the RAS group (manifested by the mobilization of the antioxidant carnosine) to a “structural maintenance”-prioritized synthesis mode in the RRCS group (manifested by LysoPC-mediated membrane lipid remodeling). This optimized allocation of metabolic resources is speculated to greatly improve the efficiency of nutrient deposition into the muscle. Ultimately, this shift in metabolic allocation strategy is associated with the increased proportion of high-quality fatty acids (such as DHA and EPA) in the muscle, as well as the significantly enhanced muscle hardness and springiness at the physical level by reducing tissue moisture content and increasing fiber density. This metabolic reallocation, orchestrated along the gut-hepatopancreas-muscle axis, may serve as a fundamental molecular basis for the superior muscle quality achieved under the rice–crayfish co-culture model.

Despite the insightful findings derived from the systematic comparison of the two culture modes, certain limitations of the present study should be explicitly acknowledged. First, regarding the inherent environmental differences between natural habitats and artificial systems, since the paddy field and the recirculating aquaculture system have fundamental differences in water quality fluctuations, spatial layout, and microbial contact frequency, these habitat-specific complex factors (including various unmeasured environmental variables such as natural light intensity, weather fluctuations, and sediment micro-environments) may still introduce systematic biases, including the inherent difference in stocking density (19.1 ind/m^2^ in RAS vs. 3.75 ind/m^2^ in RRCS), that are difficult to fully isolate, even with strenuous efforts to control variables through standardized management. Consequently, our findings should be interpreted as system-level physiological outcomes associated with each aquaculture model rather than as direct causal inferences attributable to any single environmental variable. Future studies employing factorial designs or intermediate-scale semi-controlled systems will be necessary to disentangle the individual contributions of specific factors such as stocking density, spatial complexity, and dietary composition. Second, this study was conducted at a single time point (92 days). Whether the observed physiological divergences between RAS and RRCS would persist, diminish, or reverse over extended rearing remains unknown, and future longitudinal studies are needed to address this question. Third, constrained by the sample size and the setting of a single experimental site, the general applicability of the regulatory patterns revealed in this study across broader regions or different farming scales remains to be further parsed through multi-site and large-population field validations. Fourth, regarding statistical analyses, a formal a priori power analysis was not conducted to optimize sample size, nor were multiple comparison corrections applied to the non-metabolomics parameters. We acknowledge these as limitations of the current exploratory stage, which future confirmatory studies should address. Fifth, formal correlation analyses between specific environmental parameters and intestinal microbiota were not performed because the key environmental differences between RAS and RRCS are highly qualitative (e.g., spatial complexity, sediment), highlighting the need for future studies with controlled environmental gradients. Sixth, regarding microbial profiling, while computational functional prediction (PICRUSt2) provided valuable cross-omics insights, actual shotgun metagenomics was not performed. These functional predictions remain inferential and should be interpreted as hypothesis-generating rather than definitive. Future research should incorporate metagenomics and targeted mechanistic approaches (e.g., germ-free models or microbial colonization) to definitively validate the causal biological mechanisms behind the gut-hepatopancreas-muscle axis, as the current multi-omics integration remains inherently correlational. Finally, the key differential metabolites identified via untargeted LC-MS were putatively annotated (MSI Level 2) and were not further validated using pure analytical reference standards; therefore, these metabolite identities and the resulting physiological interpretations should be regarded as plausible hypotheses requiring targeted absolute quantification in future studies.

## 5. Conclusions

This study indicates that Recirculating Aquaculture Systems (RASs) and Rice–Redclaw Crayfish Co-culture Systems (RRCSs) are associated with distinctly different regulatory orientations on the physiology of red claw crayfish. The RAS mode significantly enhanced growth rates and meat yield, making it suitable for intensive production aimed at high biomass output. In contrast, the RRCS mode demonstrated superior capacity for quality regulation. This ecosystem not only endowed the crayfish with a firmer muscle texture and superior nutritional profiles but also remodeled a healthy gut microbiota to drive the directional allocation of hepatopancreatic metabolic resources toward muscle anabolism. From a practical perspective, these findings provide clear guidance for market-oriented farming strategies. Given the market positioning of red claw crayfish as a premium, high-value species, we highly recommend the RRCS mode for premium-grade production to improve muscle quality and ensure microecological resilience against potential pathogens. Conversely, the RAS model can be strategically utilized for rapid biomass turnover. Future research could explore whether introducing beneficial microbiota into RASs might ameliorate the observed dysbiosis and seamlessly combine rapid growth with improved muscle quality.

## Figures and Tables

**Table 1:** Routine water quality parameters of the two aquaculture systems during the experimental period.

Parameters	RAS	RRCS	Safe Range
Temperature (°C)	20–25	20.0–32.0	20.0–32.0
pH	7.39–8.06	7.30–7.92	7.0–8.5
DO (mg/L)	6.0–6.7	4.8–8.0	>4.0
NH_4_^+^-N (mg/L)	0.041–0.051	0.035–0.082	<0.5
NO_2_^−^-N (mg/L)	0.030–0.037	0.025–0.045	<0.1
COD (mg/L)	11.8–14.9	12.1–18.6	<30.0

**Table 2:** Effects of two different culture modes on growth performance of red claw crayfish after 92 days.

Parameters	RAS (*n* = 15)	RRCS (*n* = 15)	*p*-Value	FDR
W_0_ (g)	9.05 ± 0.20	9.05 ± 0.19	0.949	0.949
W_t_ (g)	54.95 ± 3.43	40.50 ± 1.09	<0.001	<0.001
SR (%)	80.67 ± 1.53	80.33 ± 2.52	0.854	0.854
WGR (%)	507.39 ± 36.93	347.62 ± 17.40	<0.001	<0.001
SGR (% day^−1^)	1.96 ± 0.06	1.63 ± 0.04	<0.001	<0.001
HSI (%)	7.38 ± 0.15	6.86 ± 0.13	<0.001	<0.001
MY (%)	18.78 ± 0.73	15.36 ± 0.52	<0.001	<0.001
CF (g/cm^3^)	0.035 ± 0.001	0.031 ± 0.001	<0.001	<0.001

**Table 3:** Textural properties of red claw crayfish muscle reared under two different culture modes. Resilience and cohesiveness are dimensionless ratios calculated from the force-time curves.

Parameters	RAS (*n* = 9)	RRCS (*n* = 9)	*p*-Value	FDR
Hardness (gf)	222.67 ± 45.67	280.36 ± 33.94	<0.01	0.010
Springiness (mm)	0.69 ± 0.13	0.87 ± 0.21	<0.05	0.048
Resilience	0.39 ± 0.01	0.46 ± 0.01	<0.001	<0.001
Cohesiveness	0.45 ± 0.09	0.68 ± 0.17	<0.01	0.008
Gumminess (gf)	131.73 ± 21.82	173.14 ± 33.12	<0.01	0.010
Chewiness (mJ)	102.56 ± 13.19	143.64 ± 16.82	<0.001	<0.001

**Table 4:** Proximate composition of muscle in red claw crayfish under different culture modes (%).

Index	RAS (*n* = 6)	RRCS (*n* = 6)	*p*-Value	FDR
Moisture (%)	80.05 ± 1.21	78.43 ± 1.18	<0.05	0.028
Crude protein (%)	17.38 ± 1.44	19.40 ± 1.05	<0.05	0.026
Crude lipid (%)	0.61 ± 0.05	0.72 ± 0.04	<0.01	0.006
Ash (%)	1.77 ± 0.07	1.58 ± 0.10	<0.01	0.010

**Table 5:** Amino acid profiles of muscle tissue in red claw crayfish reared under different culture modes (mg/100 g wet weight).

Amino Acid	RAS (*n* = 6)	RRCS (*n* = 6)	*p*-Value	FDR
Thr (Threonine)	308.17 ± 10.03	399.37 ± 28.22	<0.001	<0.001
Val (Valine)	540.58 ± 3.70	548.97 ± 4.92	<0.01	0.014
Met (Methionine)	206.65 ± 3.96	212.58 ± 4.23	<0.05	0.033
Phe (Phenylalanine)	566.81 ± 6.28	588.36 ± 12.76	<0.01	0.013
Ile (Isoleucine)	371.53 ± 3.94	384.27 ± 8.34	<0.01	0.014
Leu (Leucine)	799.56 ± 16.77	827.03 ± 16.71	<0.05	0.021
Lys (Lysine)	120.51 ± 7.22	108.83 ± 1.88	<0.05	0.014
ΣEAA	2913.81 ± 40.70	3069.40 ± 31.34	<0.001	<0.001
Ser (Serine)	222.35 ± 7.96	245.56 ± 12.73	<0.01	0.01
Gly (Glycine)	944.15 ± 7.45	982.91 ± 32.30	<0.05	0.033
His (Histidine)	746.08 ± 9.12	783.99 ± 10.21	<0.001	<0.001
Arg (Arginine)	2150.71 ± 23.20	2258.4 ± 69.84	<0.05	0.015
Ala (Alanine)	618.93 ± 6.19	641.41 ± 4.54	<0.001	<0.001
Tyr (Tyrosine)	424.72 ± 5.00	442.42 ± 9.23	<0.01	0.008
Pro (Proline)	178.70 ± 7.78	184.67 ± 2.55	>0.05	0.124
ΣNEAA	5285.60 ± 44.84	5539.00 ± 102.70	<0.001	0.002
ΣDAA	2554.57 ± 9.64	2655.10 ± 28.65	<0.001	<0.001
ΣTAA	8199.41 ± 31.76	8608.40 ± 118.86	<0.001	0.001

**Table 6:** Muscle fatty acid composition of red claw crayfish reared in different culture systems (%).

Fatty Acids	RAS (*n* = 6)	RRCS (*n* = 6)	*p*-Value	FDR
(C10:0)	0.021 ± 0.002	0.014 ± 0.001	<0.001	<0.001
(C12:0)	0.063 ± 0.002	0.051 ± 0.001	<0.001	<0.001
(C14:0)	0.922 ± 0.01	0.613 ± 0.005	<0.001	<0.001
(C16:0)	18.911 ± 0.168	15.077 ± 0.215	<0.001	<0.001
(C18:0)	7.387 ± 0.252	8.448 ± 0.275	<0.001	<0.001
(C20:0)	0.133 ± 0.002	0.086 ± 0.004	<0.001	<0.001
(C22:0)	0.219 ± 0.012	0.232 ± 0.014	0.091	0.091
ΣSFA	27.656 ± 0.378	24.522 ± 0.346	<0.001	<0.001
(C14:1)	0.007 ± 0.001	0.040 ± 0.002	<0.001	<0.001
(C16:1)	6.047 ± 0.067	4.101 ± 0.165	<0.001	<0.001
(C18:1)	20.134 ± 0.042	31.092 ± 0.031	<0.001	<0.001
(C20:1)	0.757 ± 0.012	1.695 ± 0.038	<0.001	<0.001
(C22:1)	0.161 ± 0.006	0.0317 ± 0.006	<0.001	<0.001
ΣMUFA	27.140 ± 0.059	37.212 ± 0.194	<0.001	<0.001
C18:2 n−6	9.252 ± 0.034	6.826 ± 0.331	<0.001	<0.001
C18:3 n−6	2.378 ± 0.053	1.518 ± 0.012	<0.001	<0.001
C18:3 n−3	1.053 ± 0.011	0.465 ± 0.021	<0.001	<0.001
C20:2 n−6	0.066 ± 0.002	0.129 ± 0.002	<0.001	<0.001
C20:3 n−6	7.453 ± 0.111	3.092 ± 0.084	<0.001	<0.001
C20:4 n-6 (ARA)	8.945 ± 0.019	9.287 ± 0.065	<0.001	<0.001
C20:5 n−3 (EPA)	7.141 ± 0.303	8.771 ± 0.181	<0.001	<0.001
C22:6 n−3 (DHA)	2.068 ± 0.060	2.257 ± 0.063	<0.001	<0.001
ΣPUFA	38.697 ± 0.224	32.002 ± 0.430	<0.001	<0.001
Σ n−3PUFA	10.262 ± 0.224	11.493 ± 0.251	<0.001	<0.001
Σ n−6PUFA	28.453 ± 0.147	20.508 ± 0.337	<0.001	<0.001
Σ n−3/Σ n−6 (%)	36.092 ± 0.951	56.005 ± 1.498	<0.001	<0.001

## Data Availability

The original contributions presented in this study are included in the article and Supplementary Materials. Further inquiries can be directed to the corresponding authors.

## Supplementary Materials

The following supporting information can be downloaded at https://www.mdpi.com/article/10.3390/foods15111857/s1: Table S1. New confidence levels of compound annotations according to the Metabolomics Standards Initiative (MSI), as discussed by the Compound Identification Work Group at the 2017 annual meeting of the Metabolomics Society (Brisbane, Australia). Table S2: Detailed annotation of the differential metabolites shown in the heatmap. Table S3: Significantly differentially abundant metabolites in the hepatopancreas of Red claw crayfish (*Cherax quadricarinatus*): RRCS vs. RAS. Table S4. Relative abundances of dominant bacterial phyla in the gut of red claw crayfish under different culture systems. Table S5. Relative abundances of dominant bacterial genera in the gut of red claw crayfish under different culture systems. Table S6. Comprehensive evaluation of standardized effect sizes (Cohen’s d) for physiological, physical, and nutritional parameters of red claw crayfish (*Cherax quadricarinatus*) cultured in RRCS versus RAS. Figure S1. Overlay of total ion chromatograms (TIC) of QC samples (QC1–QC4), showing consistent retention times and peak intensities. Figure S2. Pairwise correlation heatmap of QC samples. All correlation coefficients were >0.99, indicating good analytical repeatability. Figure S3. Relative standard deviation (RSD) distribution of metabolite features in QC samples. More than 90% of features had RSD < 30%; features with RSD > 30% were excluded.
