# Supplementary material for: Comparative Effects of Recirculating and Rice-Co-Culture Systems on Growth-Quality Trade-Offs and Underlying Physiological Mechanisms in Red Claw Crayfish (Cherax quadricarinatus)
Source: Foods. 2026 May 24;15(11):1857. doi: 10.3390/foods15111857 (PMC13256386; doi:10.3390/foods15111857)

Supplementary Materials

Table S1. New confidence levels of compound annotations, as discussed by the Compound Identification work group of the Metabolomics Society at the 2017 annual meeting of the Metabolomics Society (Brisbane, Australia). The new addition refers to the 'Level 0' annotation; other levels remain as discussed by the Metabolomics Standards Initiative.

Confidence Level	Description	Minimum Data Requirements
Level 0	Unambigous 3D structure: Isolated, pure compound, including full stereochemistry	Following natural product guidelines, determination of 3D structure
Level 1	Confident 2D structure: uses reference standard match or full 2D structure elucidation	At least two orthogonal techniques defining 2D structure confidently, such as MS/MS and RT or CCS
Level 2	Probable structure: matched to literature data or databases by diagnostic evidence	At least two orthogonal pieces of information, including evidence that excludes all other candidates
Level 3	Possible structure or class: Most likely structure, isomers possible, substance class or substructure match	One or several candidates possible, requires at least one piece of information supporting the proposed candidate
Level 4	Unkown feature of insterest:	Presence in sample

Table S2. Detailed annotation of the differential metabolites shown in the heatmap.

ID	Metabolite Name	ID	Metabolite Name
MN5793.neg	Valylalanine	MN11821.neg	Soudanone C
MP10418.pos	Thr-His	MN11931.neg	Tetranor-PGDM
MN17792.neg	Osmanthuside H	MP20638.pos	Episceptrumgenin
MN3985.neg	2,5-Furandicarboxylic acid	MP20880.pos	Bufol
MP8106.pos	Meperidinic acid	MN9498.neg	Grosheimin
MP11718.pos	5-Hydroxyprimaquine	MN21915.neg	LysoPE(22:4(7Z,10Z,13Z,16Z)/0:0)
MN10926.neg	H-TRP-SER-OH	MN10374.neg	1-Dodecanesulfonic acid, 1-hydroxy-3-oxo-
MP16781.pos	Phe-Trp	MN9497.neg	[(3e,5r)-5,9-dimethyldeca-3,8-dien-1-yl]oxysulfonic acid
MP10839.pos	ile-met	MN10265.neg	Penicitrinol D
MP19904.pos	leu-leu-arg	MN8821.neg	Illudin C2
MP14787.pos	Arg-Phe	MN8734.neg	5-Hydroxy-2-(1-hydroxy-5-methyl-4-hexenyl)benzofuran
MP12894.pos	phe-gln	MP21706.pos	3alpha,7alpha,12alpha,26-Tetrahydroxy-5alpha-cholestan-27-oic acid
MN18192.neg	(5Z,7E)-(3S)-26,26,26-Trifluoro-27-nor-9,10-seco-5,7,10(19)-cholestatriene-3,25-diol	MP40417.pos	1-octadecanoyl-2-(4Z,7Z,10Z,13Z,16Z,19Z-docosahexaenoyl)-sn-

			glycero-3-phosphoethanolamine
MP40034.pos	PC(P-18:0/18:2(9Z,11E)+=O(13))	MP3384.pos	7-methylisoquinolin-1-ol
MN14811.neg	(3R,4R)-4-Amino-1-[[4-[(3-methoxyphenyl)amino]pyrrolo[2,1-f][1,2,4]triazin-5-yl]methyl]piperidin-3-ol	MN2789.neg	L-Malic acid
MN18632.neg	(1r,2s,3r,10z,14r,15s)-2-(acetyloxy)-8-hydroxy-1,5,10,14-tetramethyl-6-oxo-7,18-dioxatricyclo[13.2.1.04,8]octadeca-4,10-dien-3-yl acetate	MN4342.neg	3-HYDROXY-3-METHYLGLUTARATE
MP11387.pos	10-oxo-14-methyl-pentadecanoic acid	MP12256.pos	wogonin
MN18096.neg	7,7-Diphenyl-2-(1-imino-2-(2-methoxyphenyl)ethyl)perhydroisoindol-4-one	MN8454.neg	A-BENZYLHYDROCINNAMIC ACID
MN17995.neg	[4-(acetyloxy)-11a-hydroxy-6,6,9a-trimethyl-1-oxo-decahydro-3h-phenanthro[1,2-c]furan-3b-yl]methyl acetate	MN7292.neg	2,6-Naphthalenedicarboxylic acid
MN19449.neg	12-(acetyloxy)-3,14,17-trihydroxy-4,9,13,17-tetramethyl-5-oxo-6-oxatricyclo[11.4.0.03,7]heptadec-8-en-2-yl acetate	MP21676.pos	2-{[4-hydroxy-2-(hydroxymethyl)-5-(6-hydroxypurin-9-yl)oxolan-3-yl]oxy}-6-(hydroxymethyl)oxane-3,4,5-triol
MP12899.pos	N-(1-Deoxy-1-fructosyl)isoleucine	MN6695.neg	L-Tryptophan
MP11964.pos	Fructosylvaline	MN6226.neg	Caffeine
MP17626.pos	Sucrose	MP3760.pos	5-Phenylnorvaline
MN15270.neg	Isomaltose	MN11093.neg	Alosetron
MN9582.neg	(8betaOH,10beta)-8-Hydroxy-3-oxo-7(11)-eremophilen-12,8-olide	MP14728.pos	Kirenol

Table S3. Significantly differentially abundant metabolites in the hepatopancreas of Red claw crayfish (Cherax quadricarinatus): RRCS vs. RAS

Metabolic Pathway	P-value	Hit / Total	Up	Down
beta-Alanine metabolism	0.001	4/32	Uracil<br>D-Pantothenic acid<br>D-4'-Phosphopantothenate	Carnosine
Citrate cycle (TCA cycle)	0.004	3/20	Fumaric acid<br>L-Malic acid<br>Citrate	—
Caffeine metabolism	0.005	3/22	Xanthine<br>Xanthosine<br>Caffeine	—
Glycine, serine and threonine metabolism	0.007	4/48	L-Tryptophan;<br>Sarcosine;<br>Dihydroxypropanoic acid<br>(2-hydroxyethyl) trimethylammonium	—
Arachidonic acid metabolism	0.008	5/79	Prostaglandin H2<br>Troxilin B3	Colfosceril palmitate

			Hydroxy-eicosatetraenoic acid<br>epoxy-eicosatrienoic acid	
Alanine, aspartate and glutamate metabolism	0.009	3/28	Fumaric acid<br>Citrate	N-Acetyl-L-aspartic acid
Pantothenate and CoA biosynthesis	0.011	3/30	Uracil<br>D-Pantothenic Acid<br>D-4'-Phosphopantothenate	—
Glycerophospholipid metabolism	0.011	4/56	(2-hydroxyethyl) trimethylammonium<br>LysoPC(P-18:1(9Z)/0:0)<br>LysoPC(P-18:1(9Z)/0:0)<br>LysoPC(22:4(7Z,10Z,13Z,16Z)/0:0	Colfosceril palmitate
D-Amino acid metabolism	0.023	4/69	D-Ornithine	Diaminopimelic acid<br>Ala-Ala<br>Cadaverine
Tryptophan metabolism	0.042	4/83	L-Tryptophan<br>3-Indoleacetamide<br>N.omega.-methyltryptamine<br>Picolinic acid	—

Table S4. Relative abundances of dominant bacterial phyla in the gut of red claw crayfish under different culture systems

Taxon	RRCS	RAS
p_Firmicutes_D	4.72×10^{-1}	2.52×10^{-1}
p_Proteobacteria	1.02×10^{-1}	2.65×10^{-1}
p_Firmicutes_A	1.82×10^{-1}	9.00×10^{-4}
p_Fusobacteriota	6.72×10^{-3}	7.31×10^{-2}
p_Planctomycetota	1.50×10^{-4}	7.82×10^{-2}
p_Cyanobacteria	3.33×10^{-5}	7.77×10^{-2}
p_Bacteroidota	6.62×10^{-2}	1.67×10^{-5}
p_Actinobacteriota	1.50×10^{-3}	3.14×10^{-2}
p_Chloroflexota	1.17×10^{-4}	2.81×10^{-2}
p_Gemmatimonadota	0	1.18×10^{-2}

Table S5. Relative abundances of dominant bacterial genera in the gut of red claw crayfish under different culture systems

Taxon	RRCS	RAS
g_Hepatoplasma	1.65×10^{-1}	1.46×10^{-2}
g_Vibrio	5.00×10^{-5}	9.22×10^{-2}
g_Hypnocyclicus	1.67×10^{-5}	6.56×10^{-4}
g_Chryseobacterium	6.15×10^{-2}	3.50×10^{-4}
g_Tabrizicola	4.25×10^{-2}	6.67×10^{-5}
g_IMCC34051	4.21×10^{-2}	0
g_NIES-981	3.61×10^{-2}	0
g_Aestuariivirga	2.86×10^{-2}	1.67×10^{-5}
g_Hyphomicrobium_A	1.62×10^{-2}	1.67×10^{-5}
g_Mariniblastus	1.47×10^{-2}	0

Table S6. Comprehensive evaluation of standardized effect sizes (Cohen's d) for physiological, physical, and nutritional parameters of red claw crayfish *(Cherax quadricarinatus)* cultured in RRCS versus RAS.

Parameters	Effect Size (Cohen's *d*)	Parameters	Effect Size (Cohen's *d*)
W_0 (g)	0.00	Tyr (Tyrosine)	2.38
W_t (g)	5.68	Pro (Proline)	1.03
SR (%)	0.16	ΣNEAA	3.20
WGR (%)	5.53	ΣDAA	4.70
SGR (% day^{-1})	6.47	ΣTAA	4.70
HSI (%)	3.70	(C10:0)	4.43
MY (%)	5.40	(C12:0)	7.59
CF (g/cm^3)	4.00	(C14:0)	39.08
Hardness (gf)	1.43	(C16:0)	19.87
Springiness (mm)	1.03	(C18:0)	4.02
Resilience	7.00	(C20:0)	14.87
Cohesiveness	1.69	(C22:0)	1.00
Gumminess (gf)	1.48	ΣSFA	8.65
Chewiness (mJ)	2.72	(C14:1)	20.88
Moisture (%)	1.36	(C16:1)	15.45
Crude protein (%)	1.60	(C18:1)	297
Crude lipid (%)	2.43	(C20:1)	33.38
Ash (%)	2.20	(C22:1)	21.55
Thr (Threonine)	4.31	ΣMUFA	70.28
Val (Valine)	1.93	C18:2n-6	10.31
Met (Methionine)	1.45	C18:3n-6	22.39
Phe(Phenylalanine)	2.14	C18:3n-3	35.08
Ile (Isoleucine)	1.95	C20:2n-6	31.50
Leu (Leucine)	1.64	C20:3n-6	44.31
Lys (Lysine)	2.21	C20:4n-6(ARA)	7.15
ΣEAA	4.28	C20:5n-3(EPA)	6.53
Ser (Serine)	2.19	C22:6n-3(DHA)	3.07
Gly (Glycine)	1.65	ΣPUFA	19.53
His (Histidine)	3.92	Σn-3PUFA	5.17
Arg (Arginine)	2.07	Σn-6PUFA	30.55
Ala (Alanine)	4.14	Σn-3/Σn-6(%)	15.87

Figure S1. Overlay of total ion chromatograms (TIC) of QC samples (QC1–QC4), showing consistent retention times and peak intensities.

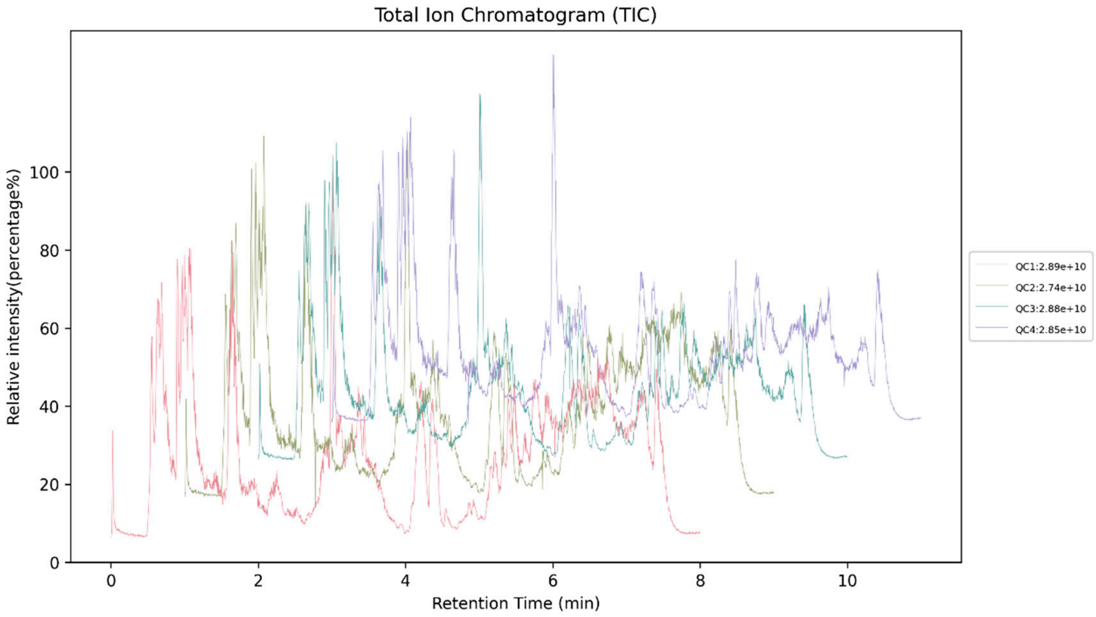

Figure S2. Pairwise correlation heatmap of QC samples. All correlation coefficients were >0.99, indicating good analytical repeatability.

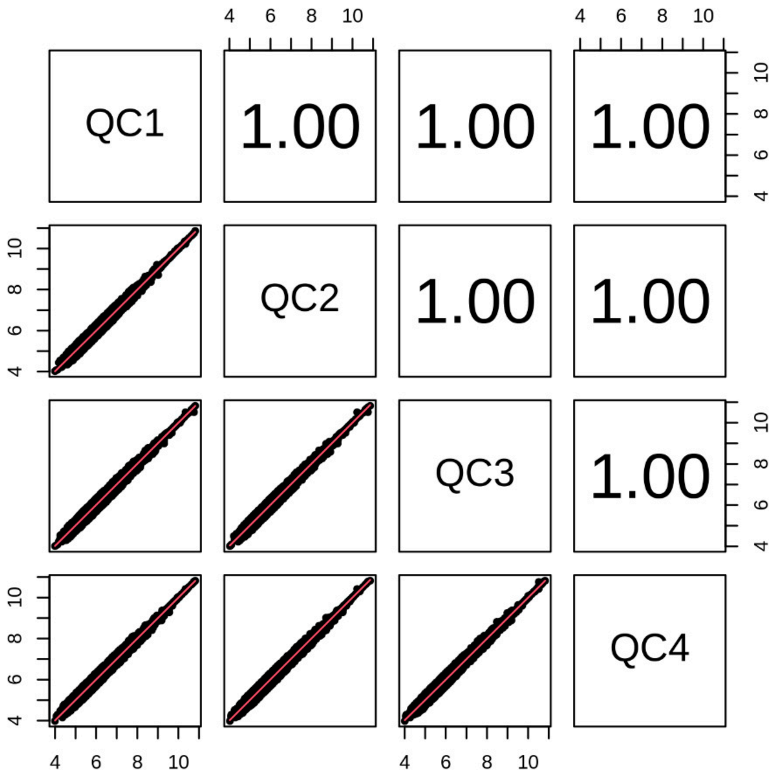

Figure S3. Relative standard deviation (RSD) distribution of metabolite features in QC samples. More than 90% of features had RSD < 30%; features with RSD > 30% were excluded.

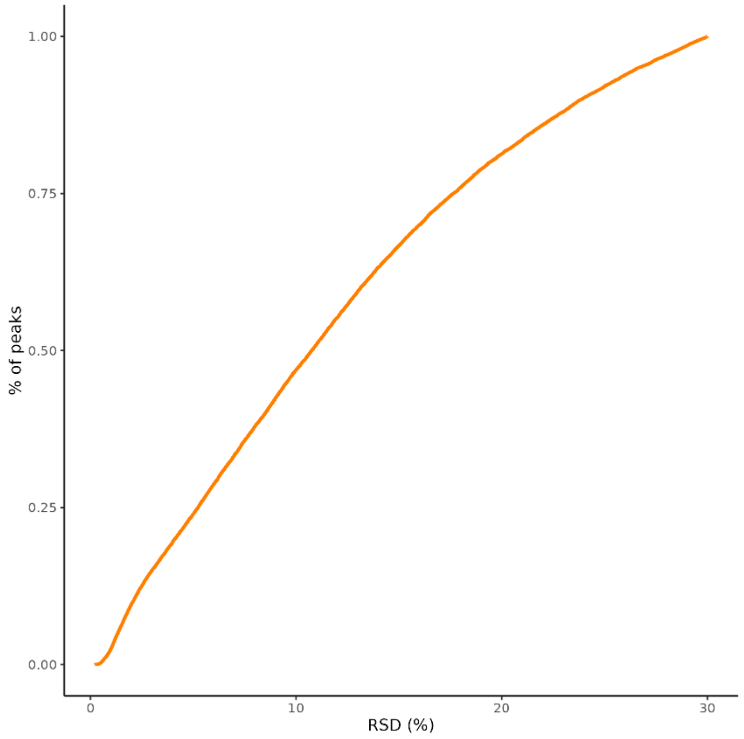

Supplement: Supplementary file 1 [file foods-15-01857-s001.zip › foods-4234829-supplementary.pdf]
